# TCN1 is a potential prognostic biomarker and correlates with immune infiltrates in lung adenocarcinoma

**DOI:** 10.1186/s12957-022-02556-8

**Published:** 2022-03-14

**Authors:** Haining Li, Liping Guo, Zhigang Cai

**Affiliations:** Department of Respiratory and Critical Care Medicine, The Second Hospital of Hebei Medical University, No.215 Heping West Road, Shijiazhuang, Hebei Province China

**Keywords:** TCN1, LUAD, Prognosis, Immune infiltrates

## Abstract

**Background:**

Around the world, lung cancer is the leading cause of cancer-related death. Lung adenocarcinomas are among the most common diagnosed forms of lung cancer, whose overall survival has not improved significantly, which makes finding an effective therapeutic target vital. Transcobalamin (TCN1) is a vitamin B12-binding protein which regulates cobalamin homeostasis. In tumor tissues, TCN1 is expressed highly, and its expression is correlated with cancer aggressiveness and poor prognosis according to recent studies and bioinformatic analyses. However, its effect on lung adenocarcinoma (LUAD) is unknown.

**Methods:**

We evaluated whether TCN1 shows diagnostic and prognostic value in LUAD using bioinformatic analysis. In particular, various databases and analysis tools were used to determine TCN1’s relationship with LUAD, including TCGA, GTEx, GEO, STRING, and TISIDB.

**Results:**

As compared to normal lung tissue, the level of TCN1 expression in LUAD tissues was significantly higher (*P* < 0.001). TCN1 also had a good ability to distinguish lung adenocarcinoma from non-lung adenocarcinoma samples [area under the curve (AUC) = 0.788]. According to univariate Cox statistics, high expression levels of TCN1 correlate with poor overall survival (OS) in LUAD (*P* < 0.001). Moreover, based on a multivariate Cox analysis, TCN1 expression was independently correlated with OS (*P* = 0.011). GO/KEGG and GSEA indicated enrichment in epidermal cell differentiation (*P* < 0.0005), keratinocyte differentiation (*P* < 0.0005), neuroactive ligand–receptor interaction (*P* < 0.0005), epithelial–mesenchymal transition (*P* = 0.029, FDR = 0.023) and TNFA signaling via NFKB (*P* = 0.029, FDR = 0.023). Furthermore, TCN1 is associated with immune infiltration based on an analysis of immune cell infiltration.

**Conclusions:**

In summary, TCN1 could be used as a prognostic and diagnostic biomarker and provide deeper perspectives for the development of therapies and prognostic markers in LUAD.

## Introduction

Globally, lung cancer is one of the most common causes of cancer-related death [1]. About 85% of lung cancer cases are non-small cell lung cancer (NSCLC), of which lung adenocarcinoma is the most common pathological subtype [2]. During the past decade, the diagnosis and treatment of lung cancer have been developed continuously, especially the emergence of targeted therapy, which has led to the extension of many patients’ lives. However, the OS for lung adenocarcinoma has not improved substantially, due to the lack of effective therapeutic targets [3]. Therefore, it is urgent to identify novel molecular mechanisms and effective therapeutic targets for LUAD.

TCN1 [haptocorrin or vitamin B12 (cobalamin) R binder] is one of the three transport proteins that binds vitamin B12 and is present in human serum and various biological fluids [4]. It plays multiple roles in keeping the basic functions of cell metabolism and proliferation, particularly in hematopoiesis and neural development [5]. Upregulation of TCN1 has been reported in the cytoplasm of tumor tissues, and TCN1 is overexpressed in some malignant tumors and associated with tumor proliferation and metastasis, such as colon cancer, hypopharyngeal squamous cell cancer, breast cancer, and gastric cancers [6–8]. The overexpression of TCN1 in tumor tissues causes tumorigenesis and poor biological behavior [9]. Recently, a study on clinicopathological analysis and prognostic by Zhu et al. has reported that TCN1 may be a vital oncogene for colorectal cancer [10]. However, the expression of TCN1 in LUAD and its clinical significance remain unclear.

During this study, we first assessed the correlation between TCN1 expression and the prognosis of LUAD patients from the GEO (Gene Expression Omnibus) database and TCGA (The Cancer Genome Atlas) database. Furthermore, our study examined the relationship between TCN1 mRNA levels and tumor-infiltrating immune cells. Based on these results, we can better understand TCN1’s role in LUAD and the immune response against tumors.

## Results

### LUAD patients’ clinical characteristics

From the TCGA database, we downloaded clinical and gene expression data of 535 tumors and 59 normal samples, including patients’ age, gender, T classification, M classification, N classification, and pathologic stage (Table 1).

**Table 1:** Clinical characteristics of the LUAD patients

Characteristic	Levels	Overall
*n*		535
Age, *n* (%)	≤65	255 (49.4%)
	> 65	261 (50.6%)
Gender, *n* (%)	Female	286 (53.5%)
	Male	249 (46.5%)
T stage, *n* (%)	T1	175 (32.9%)
	T2	289 (54.3%)
	T3	49 (9.2%)
	T4	19 (3.6%)
N stage, *n* (%)	N0	348 (67.1%)
	N1	95 (18.3%)
	N2	74 (14.3%)
	N3	2 (0.4%)
M stage, *n* (%)	M0	361 (93.5%)
	M1	25 (6.5%)
Pathologic stage, *n* (%)	Stage I	294 (55.8%)
	Stage II	123 (23.3%)
	Stage III	84 (15.9%)
	Stage IV	26 (4.9%)
Smoker, *n* (%)	No	75 (14.4%)
	Yes	446 (85.6%)

### TCN1 expression in different tumors and in LUAD patients

**Fig. 1:**
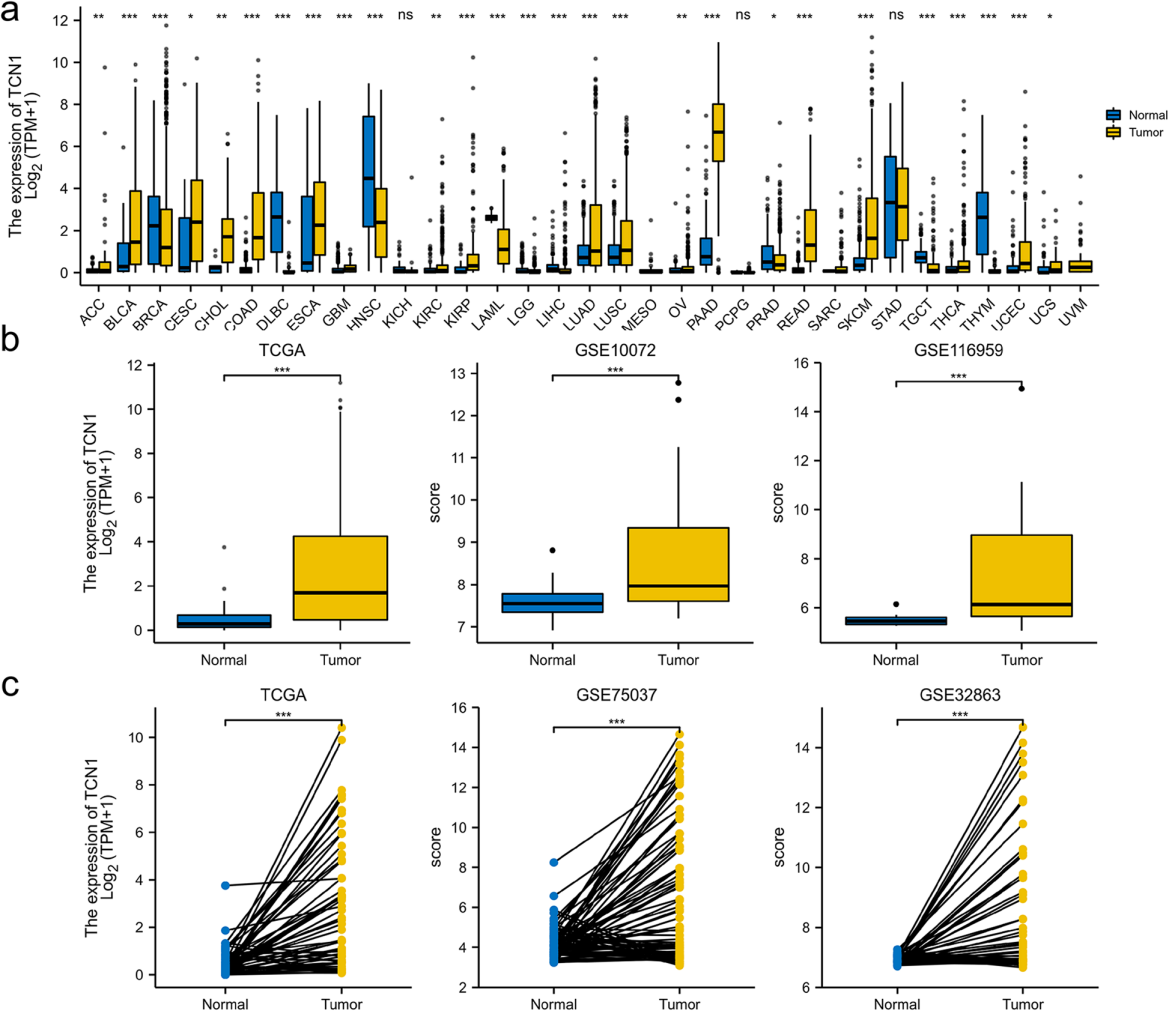
TCN1 expression status in tumors. TCN1 mRNA expression in different types of tumor tissues and normal tissues based on the TCGA database and GTEx database (**a**). TCN1 mRNA expression in LUAD tissues and normal tissues based on the TCGA database and GEO database (**b**). TCN1 mRNA expression in LUAD tissues and adjacent tissues based on the TCGA database and GEO database (**c**). *P < 0.05, **P < 0.01, and ***P < 0.001

First, we examined the expression of TCN1 in various tumor and normal tissue types using the TCGA database and GTEx database. As a result, we concluded the expression of TCN1 was significantly enhanced compared to normal tissues in colon adenocarcinoma (COAD), bladder urothelial carcinoma (BLCA), in cholangiocarcinoma (CHOL), kidney renal papillary cell carcinoma (KIRP), uterine corpus endometrial carcinoma (UCEC), lung squamous cell carcinoma (LUSC), and lung adenocarcinoma (LUAD). On the contrary, the expression of TCN1 was significantly lower than that in normal control tissues in lymphoid neoplasm diffuse large B cell lymphoma (DLBC), breast invasive carcinoma (BRCA), liver hepatocellular carcinoma (LIHC), testicular germ cell tumors (TDCT), acute myeloid leukemia (LAML), and head and neck squamous cell carcinoma (HNSC) (Fig. 1a). Based on TCGA and GEO data, we analyzed TCN1 transcription levels. A significant upregulation of TCN1 mRNA was found in LUAD tissues compared to normal tissues (*P* < 0.001) (Fig. 1b). The expression of TCN1 mRNA was significantly elevated in LUAD as compared to adjacent tissues (*P* < 0.001) (Fig. 1c).

### TCN1 expression and diagnostic value in LUAD

**Fig. 2:**
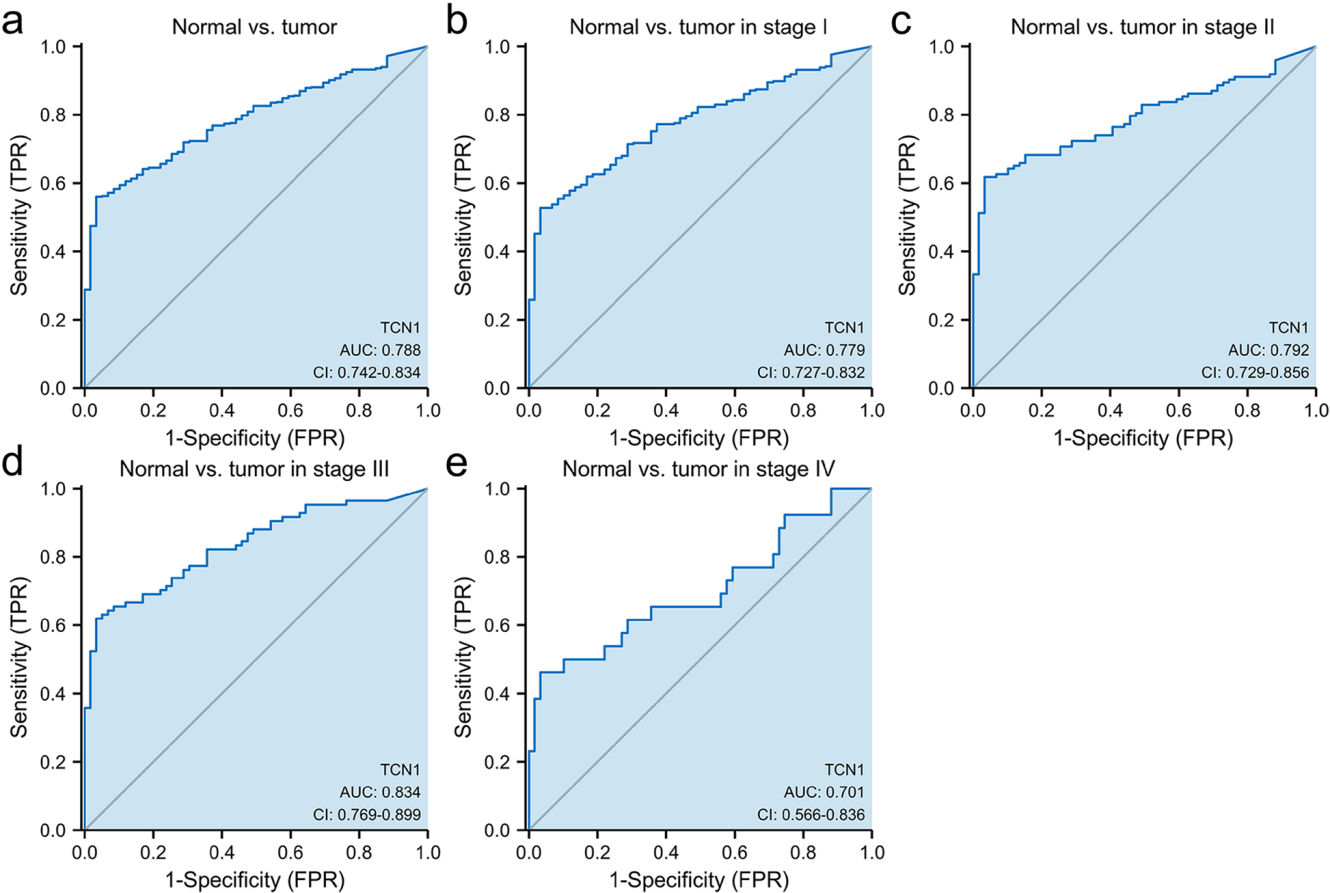
ROC curve of TCN1 mRNA expression in LUAD cohort. ROC curve of TCN1 mRNA expression in normal and tumor (**a**). Subgroup analysis for stages I, II, III, and IV, respectively (**b**–**e**)

In our study, we evaluated the diagnostic value of TCN1 mRNA expression using ROC curves. The results indicated that the area under the curve (AUC) of TCN1 was 0.788. Also, we examined the expression of TCN1 mRNA at various stages, with AUC values of 0.779, 0.788, 0.834, and 0.701 for stages I, II, III, and IV, respectively (Fig. 2).

### TCN1 expression is independently associated with a poorer outcome in patients with LUAD

**Fig. 3:**
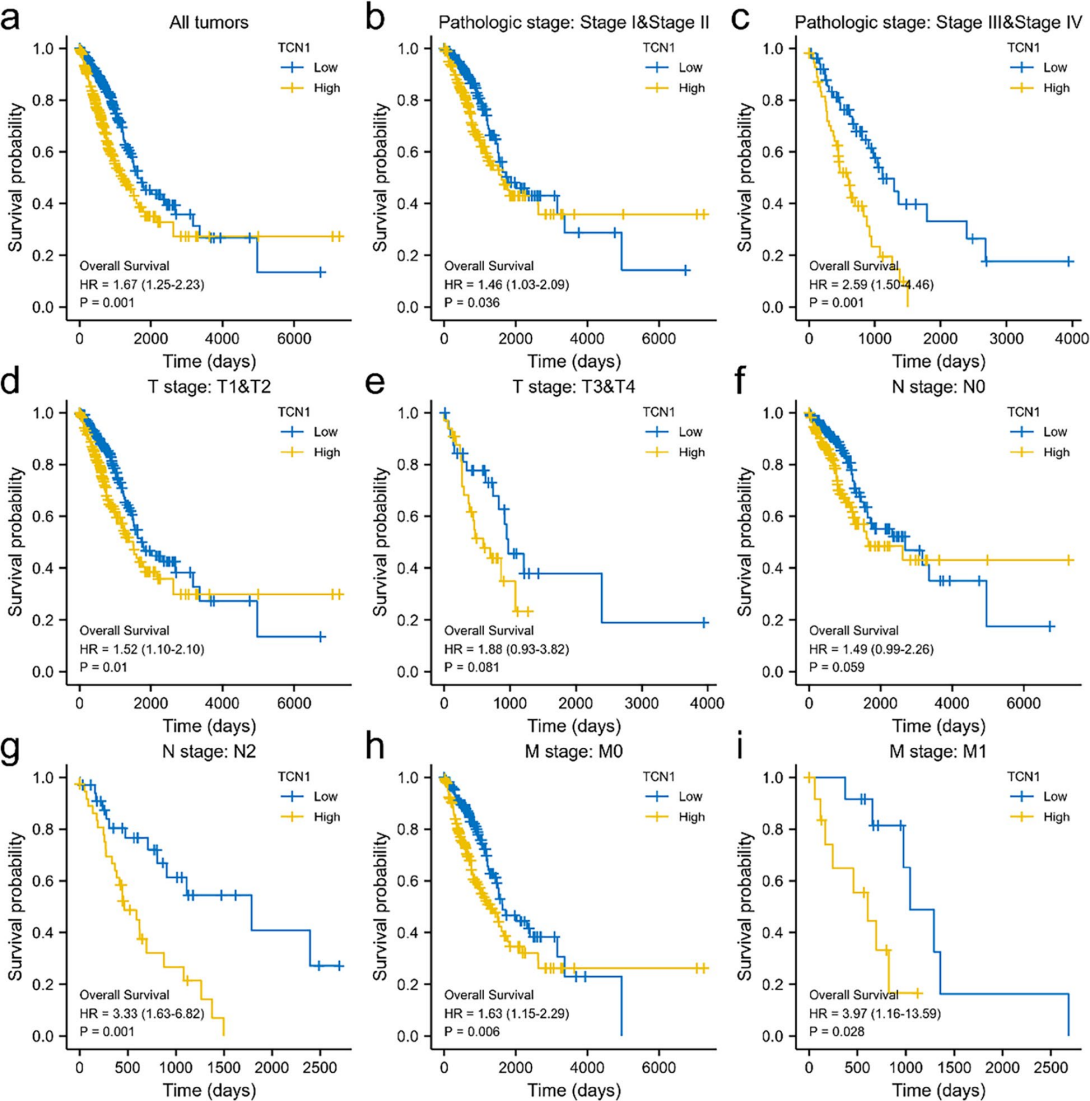
Overall survival analysis with TCN1 mRNA expression. Kaplan-Meier curves for overall survival in LUAD for all cases (**a**), pathologic stages I and II (**b**), pathologic stage III and IV (**c**), T1 and T2 (**d**), T3 and T4 (**e**), N0 (**f**), N2 (**g**), M0 (**h**), M1 (**i**)

**Fig. 4:**
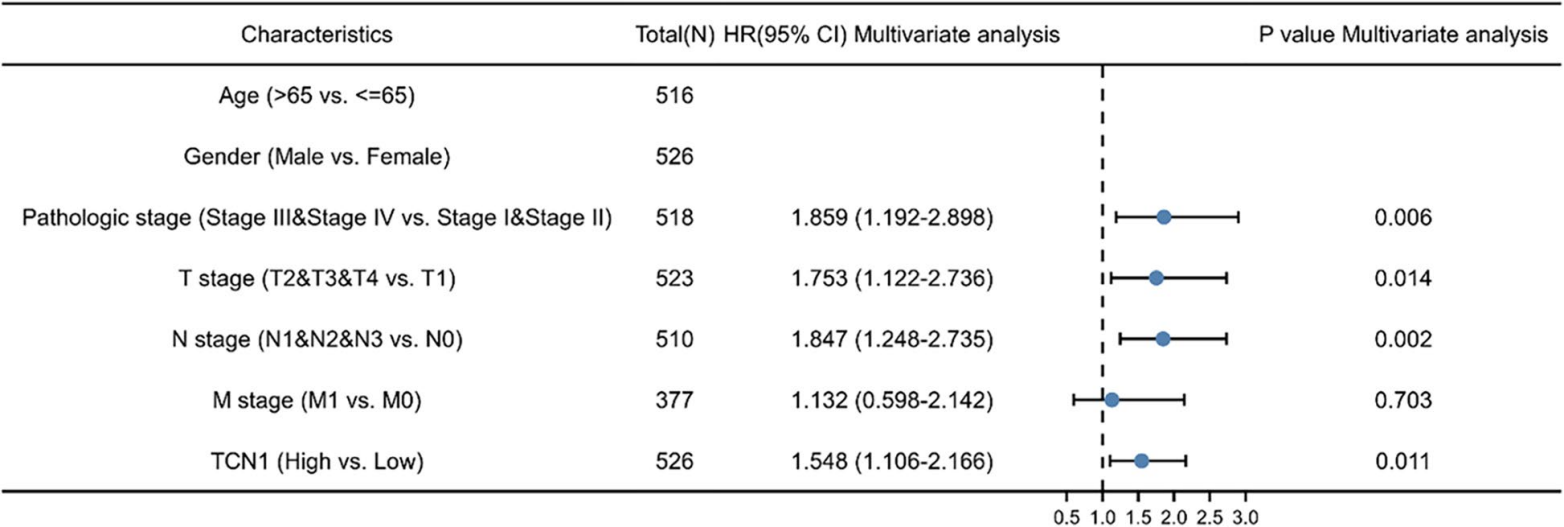
Univariate and multivariate regression analysis of TCN1 and other clinicopathologic parameters with OS in LUAD patients

Kaplan-Meier analysis indicates that high TCN1 mRNA expression was associated with poor OS (*P* = 0.001). Subgroup analysis indicated that overexpression of TCN1 mRNA significantly affected the OS in LUAD cases of pathologic stages I and II (*P* = 0.036), pathologic stages III and IV (*P* = 0.001), T1 and T2 (*P* = 0.01), N2 (*P* = 0.001), M0 (*P* = 0.006), and M1 (*P* = 0.028), respectively (Fig. 3). The univariate analysis revealed that higher TCN1 mRNA expression, T stage, N stage, M stage, and pathologic stage were correlated with OS (Table 2). According to the multivariate analysis, the mRNA expression of TCN1 was an independent risk factor for OS in LUAD (Table 2, Fig. 4).

**Table 2:** Correlations between OS and mRNA expression of TCN1 analyzed by univariate and multivariate Cox regression

Characteristics	Total *N*	Univariate analysis		Multivariate analysis	
		Hazard ratio (95% CI)	*P* value	Hazard ratio (95% CI)	*P* value
Age	516	1.223 (0.916–1.635)	0.172		
Gender	526	1.070 (0.803–1.426)	0.642		
Pathologic stage	518	2.664 (1.960–3.621)	**< 0.001**	1.859 (1.192–2.898)	**0.006**
T stage	523	1.728 (1.229–2.431)	**0.002**	1.753 (1.122–2.736)	**0.014**
N stage	510	2.601 (1.944–3.480)	**< 0.001**	1.847 (1.248–2.735)	**0.002**
M stage	377	2.136 (1.248–3.653)	**0.006**	1.132 (0.598–2.142)	0.703
TCN1	526	1.669 (1.249–2.231)	**< 0.001**	1.548 (1.106–2.166)	**0.011**

### Functional inference of TCN1 in LUAD

**Fig. 5:**
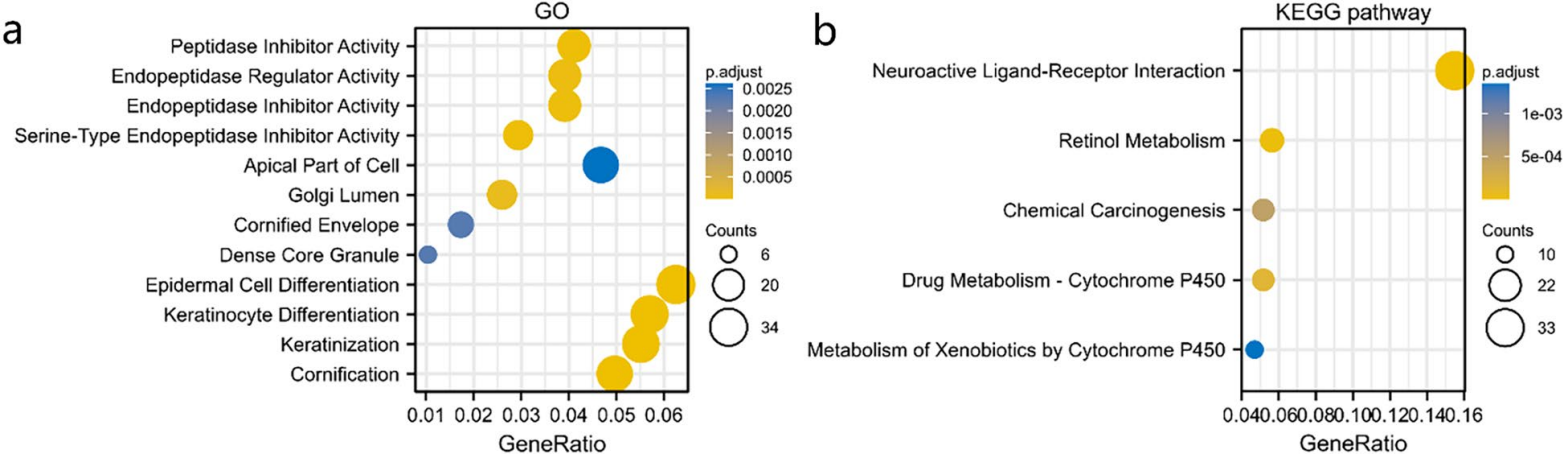
Enrichment analysis of TCN1 expression-correlated DEGs in LUAD. GO enrichment (**a**) and KEGG enrichment analysis (**b**) by TCN1 expression-correlated DEGs

GO term annotation indicated that co-expressed genes of TCN1 join mainly in epidermal cell differentiation, keratinization, keratinocyte differentiation, cornification, apical part of the cell, endopeptidase regulator activity, peptidase inhibitor activity, endopeptidase inhibitor activity, serine-type endopeptidase inhibitor activity and Golgi lumen, etc. (Fig. 5a). The KEGG pathway analysis showed enrichment in retinol metabolism, neuroactive ligand–receptor interaction, chemical carcinogenesis, drug metabolism-cytochrome P450, etc. (Fig. 5b).

### GSEA identifies a TCN1-related signaling pathway

**Fig. 6:**
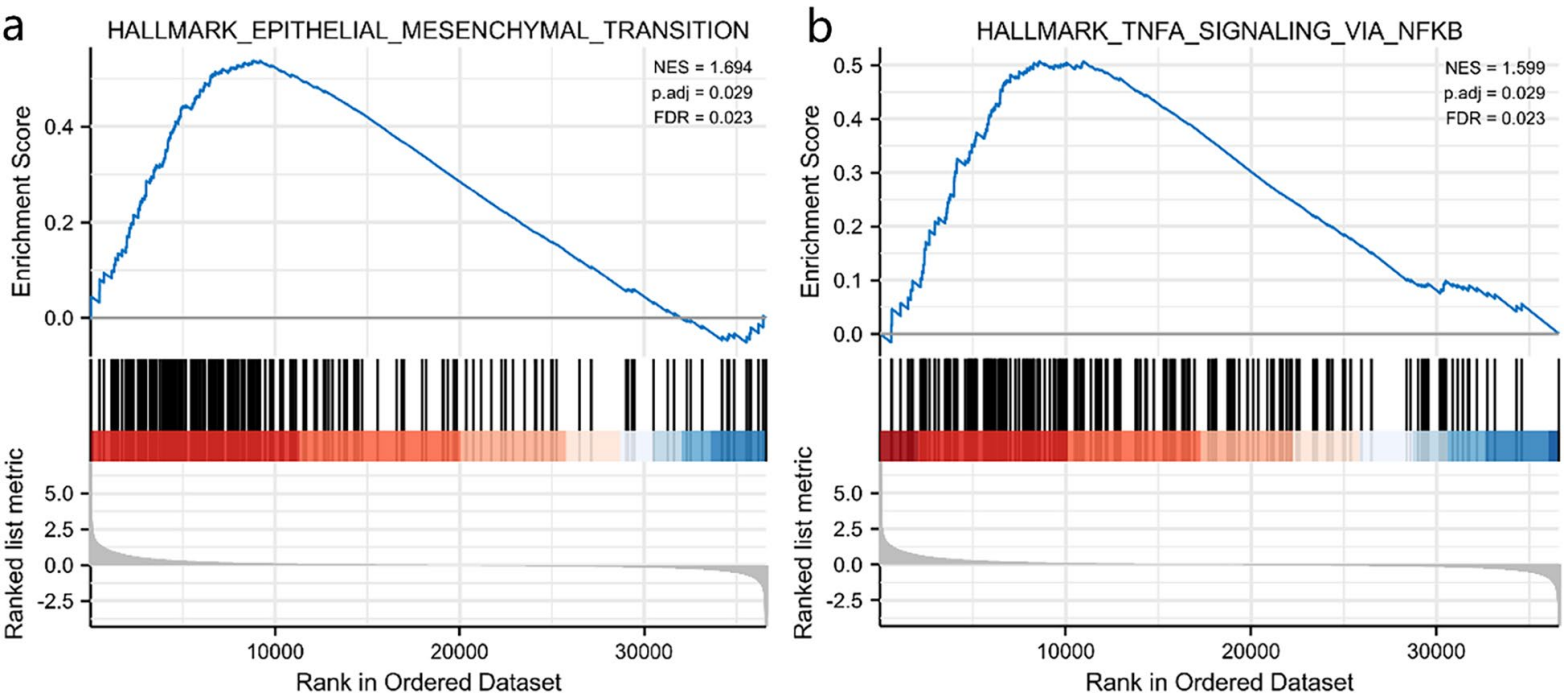
Enrichment plots by GSEA. Epithelial–mesenchymal transition (**a**). TNFA signaling via NFKB (**b**)

Signaling pathways associated with LUAD were identified using GSEA. The results showed that epithelial–mesenchymal transition and TNFA signaling via NFKB were differentially enriched in the positively correlated with TCN1 expression phenotype (Table 3, Fig. 6).

**Table 3:** Gene sets enriched in positively correlated with TCN1 mRNA expression phenotype

Gene set name	p.adj	FDR	NES
HALLMARK_EPITHELIAL_MESENCHYMAL_TRANSITION	1.694	0.029	0.023
HALLMARK_TNFA_SIGNALING_VIA_NFKB	1.599	0.029	0.023
HALLMARK_PANCREAS_BETA_CELLS	− 1.798	0.055	0.043
HALLMARK_SPERMATOGENESIS	− 1.616	0.059	0.046
HALLMARK_E2F_TARGETS	− 1.783	0.059	0.046

### Creating protein interaction networks

**Fig. 7:**
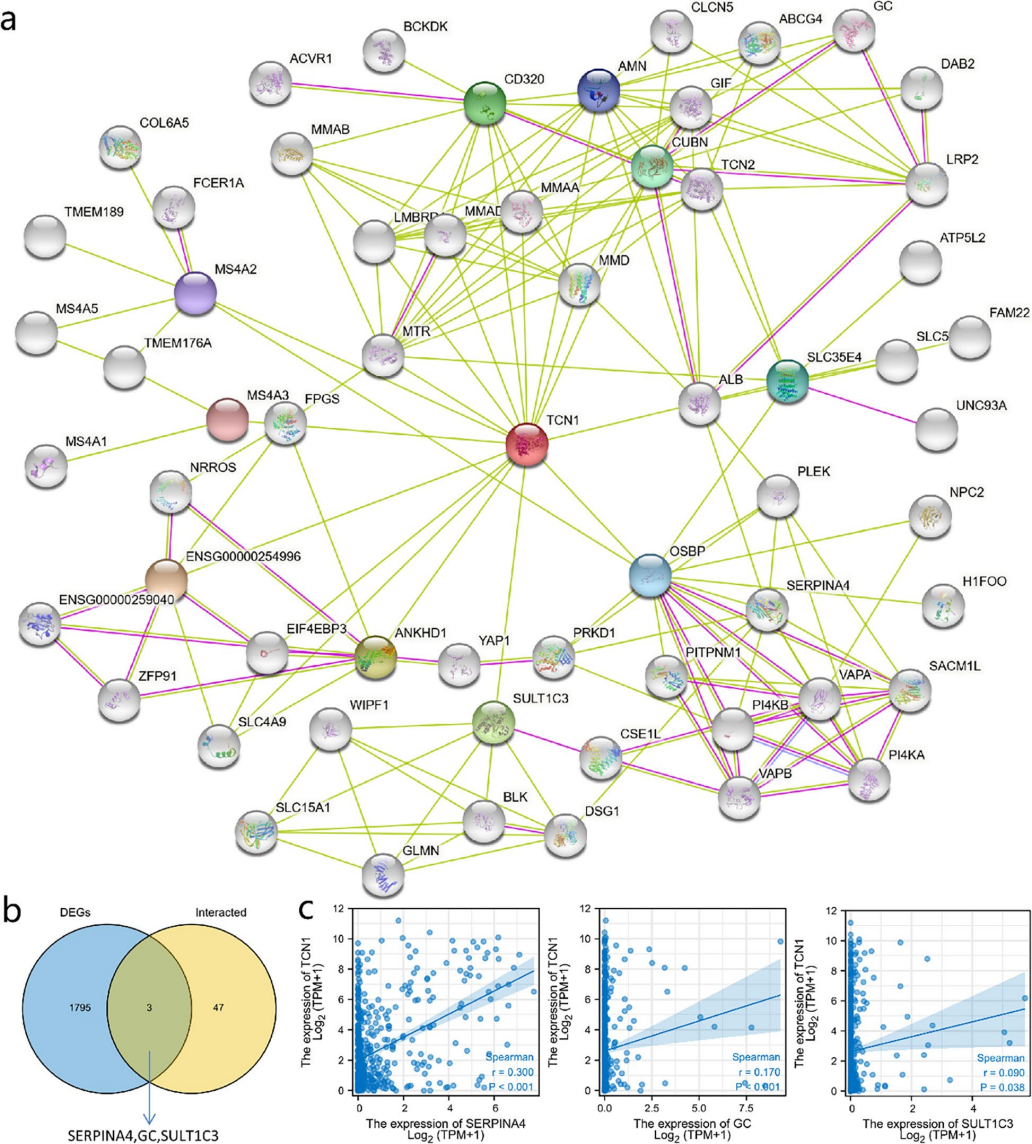
PPI network analysis of TCN1-related genes. The visualizing interaction network of TCN1-binding proteins was obtained from the STRING database (**a**). An intersection analysis of TCN1 expression correlated DEGs and TCN1-interacted genes was performed (**b**). Correlation analysis between TCN1 expression and screened common genes, including SERPINA4, GC, and SULT1C3 (**c**)

Functional interactions between proteins are essential for cancer metabolism and molecular mechanisms. Consequently, STRING was used to analyze the TCN1 protein PPI network in order to identify their interactions in LUAD progression. Using text mining and experimental evidence identification, the TCN1-binding protein interaction network was visualized (Fig. 7a). Moreover, by comparing TCN1-interacted genes with TCN1 expression-correlated DEGs, a common member was screened out such as SERPINA4, GC, and SULT1C3 (Fig. 7b). Furthermore, there was a remarkable positive association between TCN1 expression and that of SERPINA4 (*r* = 0.300, *P* < 0.001), GC (*r* = 0.170, P< 0.001), and SULT1C3 (*r* = 0.090, *P* = 0.038) (Fig. 7c).

### TCN1 expression correlates with immune infiltration levels

**Fig. 8:**
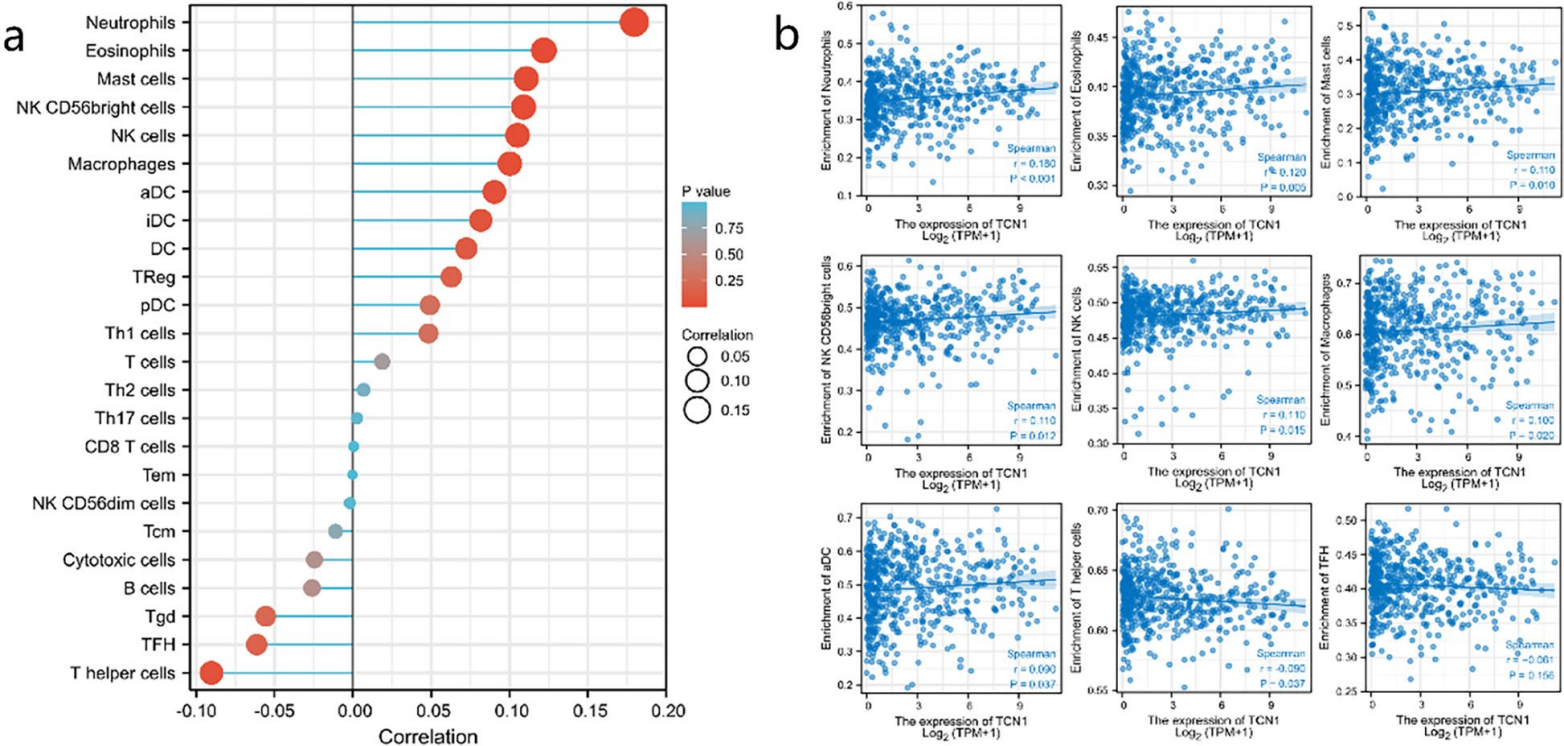
Correlation analysis of TCN1 expression with Immune infiltration in LUAD (**a**). The expression levels of TCN1 have a positive correlation with the infiltration level of neutrophil, eosinophils, mast cells, NK CD56bright cells, NK cells, macrophage, aDC, and negative correlation with the infiltration level of T helper cells and TFH (**b**)

**Fig. 9:**
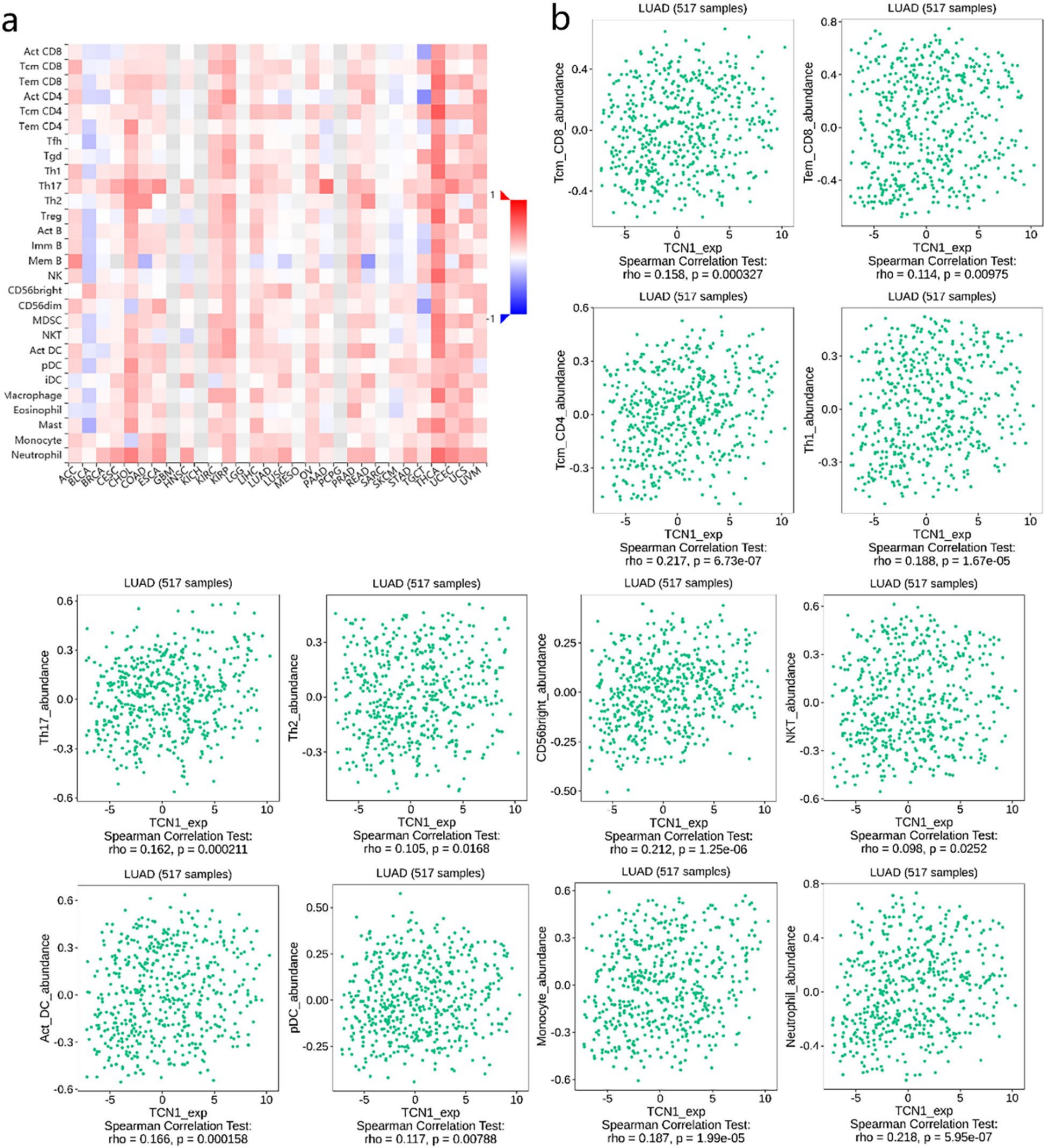
Correlation analysis of TCN1 expression with tumor-infiltrating lymphocytes (TILs) in cancer based on the TISIDB database. The landscape of relationship between TCN1 expression and TILs in multiple types of cancers (red means positive correlation and blue means negative correlation) (**a**). TCN1 expression was significantly positively associated with infiltrating levels of tcm_CD4, CD56bright, and neutrophil in LUAD (**b**)

**Fig. 10:**
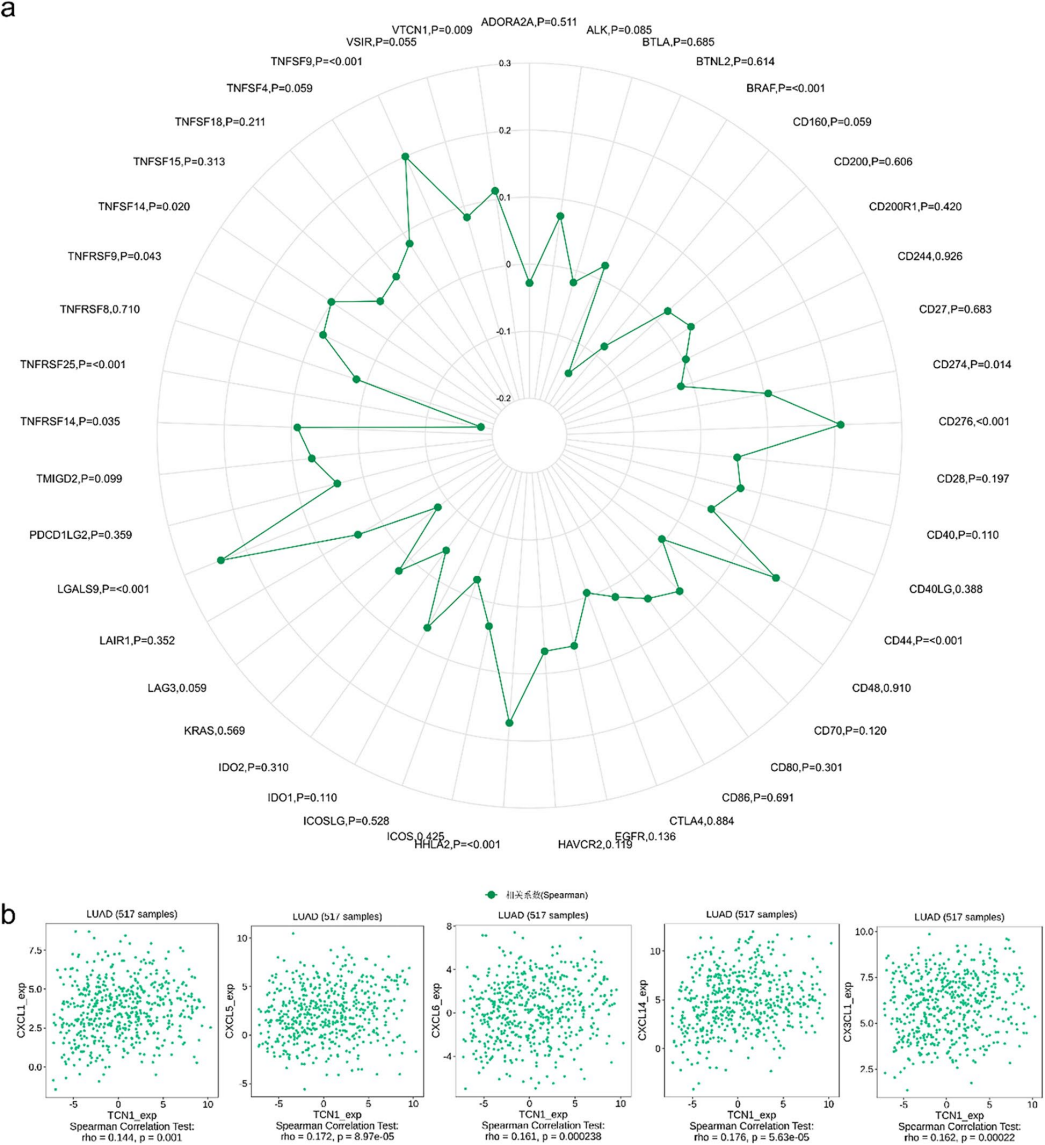
Correlation analysis of TCN1 expression with immune checkpoint genes and chemokines/chemokine receptors. Correlation analysis of TCN1 expression levels with over 40 common immune checkpoint gene levels in LUAD (**a**). TCN1 expression was positively closely related with CXCL1, CXCL5, CXCL6, CXCL14, and CX3CL1 in LUAD (**b**)

Infiltration of several types of immune cells was positively correlated with TCN1 expression, including neutrophils (*r* = 0.180, *P* < 0.001), eosinophils (*r* = 0.120, *P* = 0.005), mast cells (*r* = 0.110, *P* = 0.010), CD56bright cells (*r* = 0.110, *P* = 0.012), NK cells (*r* = 0.110, *P* = 0.015), macrophages (*r* = 0.100, *P* = 0.020), and aDC (*r* = 0.090, *P* = 0.037), while negatively correlated with the infiltration of T helper cells (*r* = − 0.090, *P* = 0.037) in LUAD (Fig. 8). Furthermore, we used TISIDB to examine the correlations between TCN1 expression and TILs. As shown in Fig. 9a, in different cancer types, TCN1 expression has been associated with TILs. Specifically, in LUAD, Multiple types of TILs were significantly correlated with TCN1 expression (Fig. 9b). Patients with many types of cancer are already benefiting from a new strategy for tumor immunotherapy—immune checkpoint inhibitors (ICIs) [11]. Following this, we compared the expression of TCN1 with over 40 other genes associated with immune control. TCN1 is closely related to the molecular targets of lung adenocarcinoma BRAF, HHLA2, and CD274 (PD-L1), especially BRAF and HHLA2. It may be related to the mechanism that elevated TCN1 leads to poor prognosis of lung adenocarcinoma (Fig. 10a). In order to understand the association between TCN1 expression and immune cell migration, we examined chemokines and chemokine receptors (Fig. 10b). According to the study results, there was a positive correlation between TCN1 expression and immune cell chemokine and receptor expression, such as CXCL1 (*r* = 0.144, *P* = 0.001), CXCL5 (*r* = 0.172, *P* = 8.97e−05), CXCL6 (*r* = 0.161, *P* = 2.38e−04), CXCL14 (*r* = 0.176, *P* = 5.63e−05) and CX3CL1 (*r* = 0.162, *P* = 2.20e−04). Considering that the expression of TCN1 increases the expression of these chemokines and chemokine receptors, high TCN1 expression may facilitate the migratory ability of immune cells.

## Discussion

While lung cancer therapy has improved, it remains one of the most malignant types of cancer with a poor five-year survival rate [12]. We urgently need to identify new biomarkers and investigate molecular mechanisms. As cancer immunotherapy has shifted paradigms in recent years, it is gradually being recognized as a promising strategy for treating some cancers [13]. The current research on immunotherapeutic strategies for lung adenocarcinoma is mainly focused on chemoradiotherapy, cancer vaccines, immune checkpoint inhibitors, and combinations with other immunotherapeutic agents, and other molecularly targeted agents. The immunotherapy field has increasingly focused on immune checkpoint inhibitor (ICI) therapy, which includes anti-PD-1 and anti-PD-L1 antibody drugs, which have evident advantages in application across cancer types and excellent clinical effectiveness [14]. However, only some patients respond well to this treatment. The key now is to discover a new biomarker that is associated with immune infiltrates and molecular mechanisms that may be responsible for the immunotherapy’s effectiveness.

As little research has been done on cancer and the TCN1 gene, we decided to perform an advanced bioinformatic analysis to investigate its potential regulatory mechanisms and biological functions in LUAD. TCN1(a member of the vitamin B12-binding protein family) is a 60–70-kDa molecular weight protein, derived from the granulocyte line [15]. TCN1, intrinsic factor, and TCN2 are involved in vitamin B12 homeostasis. TCN1 protein binds to VB12 in food protein in the stomach to protect VB12 from gastric acid. At the same time, VB12 is transported from the stomach to the duodenum, where it can be released by its own enzymatic reaction, and the intrinsic factor of VB12 in the duodenum is r-binding protein family members (intrinsic factor, IF) binding [16] for the next step of transport, and finally, VB12 is transported to plasma by TCN2 (transcobalamin II), while TCN1 is degraded by trypsin in the duodenum. TCN1 and the other two VB12-binding proteins have similar spatial structures, and each protein binds only one VB12 molecule. The difference is that TCN1 has a strong binding ability with VB12 and has a wide recognition of the range of VB12 binding sequence. In addition to participating in the transport of VB12 in extracellular body fluids, TCN1 protein can also bind to vitamin B12 in breast milk [17] and participate in the uptake of VB12 in plasma by cells by binding to the sialoglycan receptor [4]. However, the specific mechanism of the combination is still unclear. Studies have shown that 80% of VB12 in peripheral blood exists in the form of binding with TCN1 protein. As we all know, VB12 is a ubiquitous coenzyme, mainly involved in DNA synthesis and playing an important role in maintaining nervous system function, hematopoiesis, cell division, and cell metabolism [18]. Rapidly dividing malignant tumor cells also require certain vitamins, including VB12, folic acid, biotin, and riboflavin, to promote their growth and survival. A higher serum level of TCN1 and vitamin B12 has been reported in cancer patients [19]. Furthermore, it has been well established that high serum levels of vitamin B12 are associated with malignant hematological diseases such as chronic acute leukemia and myeloid leukemia, which are caused by granulocyte proliferation releasing TCN1 [15, 20]. The expression of TCN1 was also reported to be increased in hepatocellular carcinoma and immunodeficiency virus (HIV) seropositive patients [21, 22]. Also, there was a better correlation between TCN1 and progression of gastric cancer than vitamin B12 [23]. Liu et al. recently identified TCN1 expression can predict neoadjuvant chemosensitivity and is a negative prognostic biomarker in colon cancer [6]. Wang et al. found that low TCN1 expression might be a potential prognostic biomarker for predicting neoadjuvant chemotherapy sensitivity and clinical outcome in local advanced hypopharyngeal squamous cell carcinoma patients [7]. In this study, we analyzed TCN1 differential expression, diagnostic value, and prognostic value, and we also analyzed the association with tumor immune infiltration in LUAD. We found that TCN1 expression was highly elevated in LUAD, compared to normal tissues. According to our findings, TCN1 may play a vital role in LUAD development. Our findings are in accordance with previous research regarding the TCN1 role in other cancers. We found that the expression of TCN1 had certain diagnostic value in different stages of LUAD by ROC curve. Survival analysis revealed that high TCN1 expression was correlated with poor overall survival in LUAD. Our results strongly indicate that TCN1 can be used as a prognostic and diagnostic biomarker for LUAD.

Furthermore, TCN1 expression is associated with a variety of immune checkpoint markers and immune cells. According to previous studies, the immune microenvironment is crucial to tumor growth and development. For growth and metastasis, tumor cells are dependent upon a complex tumor microenvironment (TME). Tumor cells are involved in the manipulation of TME components and the progression of tumors from all stages of tumorigenesis [24]. Tumor-infiltrating immune cells, such as CD8+ T cells, CD4+ T cells, B cells, tumor-associated macrophages (TAMs), are the main components of the lung cancer microenvironment [25–27]. The GSVA package of R was used to observe the tumor-infiltrating immune cell levels in relation to TCN1 expression in order to explore its possible role in the immune system. Our results show that the expression of TCN1 correlates with multiple immune markers and immune infiltration levels in LUAD. In addition, the study suggests that TCN1 expression is closely related with tcm_CD4, CD56bright, and neutrophil in LUAD. Furthermore, according to our study, TCN1 is closely related to BRAF, HHLA2, and CD274 (PD-L1), especially BRAF and HHLA2. As an important proto-oncogene, the BRAF gene is an upstream regulator of the ras-Raf-MEK-ERK pathway and participates in the mitogen-activated protein kinase (MAPK) cascade reaction, which plays an important role in regulating cell growth and reproduction. The abnormal signal of the MAPK pathway is mainly caused by RAS and BRAF mutations. When mutations occur, BRAF protein phosphorylation and downstream extracellular regulated kinases (ERK) continue to be activated, ultimately leading to the occurrence of tumors [28, 29]. HHLA2, a newly discovered member of the B7 family, is involved in the inhibitory function of T cells. Studies have confirmed that HHLA2 is highly expressed in most malignant tumor tissues, including breast cancer, lung cancer, thyroid cancer, melanoma and pancreas adenocarcinoma, ovarian cancer, liver cancer, bladder cancer, colon cancer, prostate cancer, kidney cancer, and esophageal cancer. This suggests that HHLA2 expression may be responsible for tumor progression, and HHLA2 can give tumor survival advantage by inhibiting host anti-tumor immunity [30]. In lung adenocarcinoma, there is a significant association between high TILs infiltration and HHLA2 expression after adaptation to the mutated state. This suggests that different factors may be involved in TIL recruitment, and HHLA2 expression may be at least partially involved in TIL infiltration in lung adenocarcinoma. HHLA2 binds to a variety of immune cell receptors, including T cells and antigen-presenting cells, thereby inhibiting cell proliferation and cytokine production of human CD4 and CD8 T cells [31]. According to the GSEA analysis of our study, the potential mechanism may be involved in epithelial–mesenchymal transition (EMT) and TNFA signaling via the NFKB pathway. These may be the potential mechanisms of TCN1 leading to poor prognosis of lung adenocarcinoma. Nevertheless, an experiment has not been undertaken to verify this inference, which is the limitation of our study. For complete elucidation of the biological function of TCN1 in LUAD, further basic and clinical experiments are needed.

## Conclusions

In summary, TCN1 could be used as a prognostic and diagnostic biomarker. In addition, the study suggests that TCN1 expression is closely related with tcm CD4, CD56bright, and neutrophil in LUAD. Furthermore, according to our study, TCN1, BRAF, HHLA2 have a close positive relationship, which may bring new direction and hope for immunotherapy of LUAD.

## Methods

### Date

We investigated the TCN1 expression level in various types of normal tissues and tumor in the integrated datasets combining TCGA [32] (https://portal.gdc.cancer.gov) with the GTEx (Genotype-Tissue Expression) database (https://www.gtexportal.org/ home/-index.html). TCGA are open-ended and public and do not need the approval of a local ethics committee. We obtained the profiles of RNA expression (RNA-Seq2 level 3 data; format: TPM; platform: Illumina HiSeq 2000) and clinical sample of LUAD patients from the TCGA database. TCGA included 535 LUAD samples and 59 normal lung tissue samples, which contain general information, clinicopathological details, and prognostic information. The gene expression profiling data sets (GSE10072, GSE116959, GSE75037, GSE32863) were obtained from the GEO database (https://www.ncbi.nlm.nih.gov/gds).

### Survival analysis

Based on high and low levels of TCN1 expression, patients were separated into two groups. Based on Kaplan-Meier (KM) survival curves, we constructed a prognostic classifier to determine if TCN1 expression level affects clinical outcomes of LUAD patients.

### Analyses of univariate and multivariate logistic regression

Based on univariate Cox regression analysis, we compared the OS of two cohorts of patients with LUAD and the level of TCN1 expression. Furthermore, we used multivariate analysis to determine whether TCN1 is an independent prognostic marker for OS of LUAD patients. There is statistical significance for TCN1 in Cox regression analysis, when *P* < 0.05.

### Functional enrichment analysis

Using the limma package, we explored mRNA differential expression. False positive results were corrected using the adjusted *P* value. The screening thresholds for differentially expressed genes were defined as follows: adjusted *P* < 0.05 and |log2 (fold change) | > 1 (DEGs). GO (Gene Ontology) and KEGG (Kyoto encyclopedia of genes and genomes) analyses were conducted using the clusterprofiler package to better understand TCN1 carcinogenesis. The ggplot2 package was employed for visualization. Adjusted *P* < 0.05 is considered to be statistically significant in the enrichment results.

### Gene set enrichment analysis

TCN1 mRNA expression was analyzed by R (version 3.6.3), followed by GSEA analysis using the clusterprofiler and ggplot2 package. *P* < 0.05, |ES|>1, and FDR < 0.25 were considered to be statistically significant.

### Protein protein interaction comprehensive analysis

We used the online tool STRING [33] (Search Tool for the Retrieval of Interacting Genes/Proteins) website (https://string-db.org) to investigate the protein–protein interaction (PPI) of TCN1-binding proteins. This tool included a large collection of consolidated and integrated data on protein–protein interactions. The main settings such as active interaction sources (“textmining and experiments”), meaning of network edges (“evidence”), minimum required interaction score [“Low confidence (0.400)”], and max number of interactors to show (“no more than 50 interactors”) operated. Then, we obtained the interaction networks for 50 TCN1-binding proteins. The Venn diagram was generated with R package Venn Diagram to compare the TCN1 express-related genes interacting with DEGs and TCN1. The expression of TCN1 and the genes from intersection analysis were correlated using Spearman’s correlation analysis. *P* < 0.05 was considered to be statistically significant.

### TCN1 expression and immune Infiltrates

The massively related immune cells infiltrating tumor tissue were assessed by ssGSEA (single-sample gene set enrichment analysis). We used the GSVA package of R and immune datasets to determine the infiltration level of immune cells in LUAD. The TISIDB [34] database (http://cis.hku.hk/TISIDB) is an integrated repository tool and has a significant impact in finding the interaction between tumor and immune system. With this tool, we will further explore the immune correlates of TCN1 in cancer. First, correlations between TCN1 expression and TILs across multiple types of cancer were determined via the “lymphocyte” module. Secondly, Statistical assessment was performed using a Spearman correlation to search and screen the appropriate candidates.

In addition, the correlation between TCN1 expression and immune checkpoint gene levels was analyzed using Spearman’s correlation analysis. Based on the TISIDB database, we used the “chemokine” module to investigate the relationship to determine chemokines/chemokine receptors between TCN1 and immune cell migration.

### Statistical analysis

We conducted all statistical analyses by R (3.6.3). We generated ROC curves using the pROC package. We used the chi-square test to examine the association between TCN1 mRNA expression and clinical characteristics. The prognostic value of TCN1 mRNA expression was analyzed by multivariate Cox analysis and Kaplan-Meier analysis. *P* < 0.05 represents statistical significance.

## Data Availability

The data sets during and/or analyzed during the current study are available from the corresponding author on reasonable request.

## Abbreviations

TCN1: Transcobalamin
LUAD: Lung adenocarcinoma
OS: Overall survival
NSCLC: Non-small-cell lung cancer
GTEx: Genotype-Tissue Expression
GO: Gene ontology
KEGG: Kyoto Encyclopedia of Genes and Genomes
STRING: Search Tool for the Retrieval of Interacting Genes/Proteins
PPI: Protein–protein interaction
TCGA: The Cancer Genome Atlas
KM: Kaplan-Meier
ssGSEA: Single-sample gene set enrichment analysis
